# Dying Alone Due to COVID-19: Do the Needs of the Many Outweigh the Rights of the Few—or the One?

**DOI:** 10.3389/fpubh.2020.593464

**Published:** 2020-11-30

**Authors:** Alejandra Victoria Capozzo

**Affiliations:** Institute of Virology and Technical Innovations, IVIT (CONICET-INTA), Consejo Nacional de Investigaciones Científicas y Técnicas, Buenos Aires, Argentina

**Keywords:** SARS-CoV-2, COVID-19 pandemic, healthcare, health policies, ethics

## The Situation

The COVID-19 pandemic, caused by infection with the severe acute respiratory syndrome coronavirus 2 (SARS-CoV-2), has been striking the world since it was first identified in December 2019 in China. The World Health Organization (WHO) declared the outbreak a “public health emergency of international concern” on January 30th, 2020, and recognized its pandemic status on March 11th. The pandemic has caused universal psychosocial impact (1) and global economic disruption. Discourse and measures have been discussed focused on lockdown strategies, healthcare policies (2), application of emerging treatments, accelerated clinical trials, among others. Management guidelines are continuously updated based on emerging findings (3). However, as the disease spreads through a community, suffering deepens due to strict procedures that, arguably, may be questioned from an ethical standpoint. The pandemic has sufficiently disrupted and impaired people's livelihood worldwide, and every effort to prevent any additional suffering must be made.

Many have died in isolation. Dying alone is not justifiable, even in times of infection with a pandemic virus, particularly when the impact of imposing such a radical measure on the course of the epidemic is, at least, questionable. Indeed, some have reported that the concern and anxiety of being discriminated against delay the presentation to healthcare services, and delayed diagnosis is associated with more severe disease, mainly in the elderly and in vulnerable groups (4). The fear of being alone in hospital is another barrier in seeking healthcare, and as the number of infected individuals increases within a community, more information on this “loneliness” is perceived and feared. One may argue that the situation, and the subsequent delay in seeking healthcare, would result in negative feedback that ends up sustaining disease spread in a population that is not willing to let their elders die in isolation.

Patients with severe COVID-19 are hospitalized and left alone in a room where “spaceship-dressed” health professionals visit them, speaking behind their mask and shields, trying to keep their own social distance with the patient. When patients are transferred to medium or intensive care units, they completely lose connection with their family and friends. They stay in isolation, and in many cases, eventually die, without ever having had a chance to share a final world with their beloved ones.

## Beyond the Healthcare System, COVID-19 Patients

This scene replays itself in many hospitals all over the world, worsened by the teams of exhausted healthcare workers who are overwhelmed (5) and do not receive either psychological support or at least get enough rest (6). Distressed people then take care of depressed–stigmatized sick and lonely people, worsening an already bleak scenario (7). Communication also fails. Doctors reportedly call the family once a day, to give quick feedback on the status of the patient with little room for questions. Many calls must be done in a stretched time frame and news is not always good. Healthcare workers also suffer. There is no face-to-face contact—never, nor even between caregivers and patients. For the family, the physician becomes just “a voice on the phone.” One may argue that the pandemic has demonstrated, worldwide, the massive failure of healthcare systems, in many cases, exhausted by decades of cuts to funding, training, and preparedness.

The presence of family accompanying the patients at the intensive care units has shown to be beneficial for the critical patients, their family, and the healthcare workers (8), supported also by the medical ethics literature (9). Possible options to give a solution for the loneliness of critical COVID-19 patients, proposing different alternatives for bringing company to the deathbed, including seeing the family across a shield or even pets have been proposed (10). Wakam et al. (11) published a perspective article, sharing some experiences of a group of physicians assisting COVID-19 patients. The authors reviewed day-life situations and propose to work harder in developing protocols to bridge the physical distance between the sick person and the family. They underlined the importance of being compassionate to families that are confronting the loss of their loved ones. No one deserves to die alone. There is an urgent need for creative solutions to help the patients feel some connection with their beloved ones without risking anyone's health.

There is also an urgent need to re-think healthcare systems and public health policy in times of pandemics, to debate options to improve the humane assistance to improve patients' experience. Detailed and worldwide applicable policies must be discussed and pursue the humane care of COVID-19 patients. An overwhelmed healthcare system should not be an excuse for mistreatments or, in many cases, unethical behavior.

At times when reality resembles science fiction, there is, perhaps, some wisdom in one of the lessons many of us received as children in one of the most renowned and remembered science fiction series: the needs of the many, never, ever should outweigh the needs and rights of the few (12), or the one. The needs of each one should be addressed even during the most devastating pandemic humankind has witnessed over the last century.

If we lose humanity, it will be our fault. We will not be able to blame it on the virus.

## Author Contributions

The author confirms being the sole contributor of this work and has approved it for publication.

## Conflict of Interest

The author declares that the research was conducted in the absence of any commercial or financial relationships that could be construed as a potential conflict of interest.
